# Chaotic Ising-like dynamics in traffic signals

**DOI:** 10.1038/srep01127

**Published:** 2013-01-24

**Authors:** Hideyuki Suzuki, Jun-ichi Imura, Kazuyuki Aihara

**Affiliations:** 1 Institute of Industrial Science, The University of Tokyo, Tokyo 153–8505, Japan; 2 Graduate School of Information Science and Engineering, Tokyo Institute of Technology, Tokyo 152–8552, Japan

## Abstract

The green and red lights of a traffic signal can be viewed as the up and down states of an Ising spin. Moreover, traffic signals in a city interact with each other, if they are controlled in a decentralised way. In this paper, a simple model of such interacting signals on a finite-size two-dimensional lattice is shown to have Ising-like dynamics that undergoes a ferromagnetic phase transition. Probabilistic behaviour of the model is realised by chaotic billiard dynamics that arises from coupled non-chaotic elements. This purely deterministic model is expected to serve as a starting point for considering statistical mechanics of traffic signals.

Kyoto City in Japan is famous for its grid pattern of roads, as well as its long history as the former capital. Nowadays, many vehicles use the roads, and traffic signals are placed at the crossings. The traffic signals at each crossing have essentially two states: in one state, the traffic signals for the north–south direction are green, and in the other, those for the east–west direction are green. If we relate these two states to the up and down states of an Ising spin, all of Kyoto City can be viewed as the Ising model on a two-dimensional lattice. This naive idea cannot be regarded as absolute nonsense when traffic signals are controlled depending on local traffic flows, because neighbouring signals interact indirectly through the traffic flows between them^1^,^2^. Although finding efficient control strategies for traffic signals is an important problem, their collective behaviour under such decentralised strategies has not been well understood^3^,^4^. In this paper, we reveal the physics underlying the interacting signals by showing that a simple model on a finite-size two-dimensional lattice exhibits a ferromagnetic phase transition with critical behaviour. We also report that probabilistic behaviour as an Ising-like model is realised by chaotic billiard dynamics that arises naturally from coupled non-chaotic elements. This purely deterministic model is expected to serve as a starting point for considering statistical mechanics of traffic signals.

The Ising model^5^, to which we relate Kyoto City, is an abstract model for ferromagnetism that shows phase transitions with critical behaviour. The model itself describes only probabilistic distributions of spin configurations and does not specify the underlying dynamics. The dynamics in real ferromagnetic materials may be given in terms of quantum mechanics. However, it is also intriguing to ask whether the probabilistic behaviour of the Ising model can be explained by deterministic dynamics, which is referred to as Ising dynamics in this paper. There have been two series of studies that can be regarded as following this direction.

One approach considers deterministic microcanonical dynamics, in which the spin configuration wanders deterministically on a microcanonical ensemble according to the Hamiltonian of the Ising model. In a model proposed by Creutz^6^, a demon traveling around the lattice is assumed to convey kinetic energy from spin to spin while conserving the total energy. If the demon travels deterministically, the model is deterministic. Microcanonical dynamics can also be implemented as cellular automata^7^,^8^. The Creutz cellular automaton has been shown to behave in almost the same way as Monte Carlo simulations of the original Ising model^9^.

The other approach is based on coupled map lattices (CMLs)^10^, which are an important class of dynamical systems with a large number of degrees of freedom that exhibit rich spatio-temporal nonlinear dynamics. CMLs are typically composed of chaotic elements on a lattice interacting with neighbouring elements. By defining binary symbols in the state space of each element, Ising-like spin models can be constructed on the basis of CMLs^11^,^12^. Although the microscopic behaviour of the CMLs is far from equilibrium, their macroscopic behaviour can be understood in terms of equilibrium statistical mechanics^13^,^14^,^15^,^16^,^17^,^18^.

The traffic signal model on a two-dimensional lattice that we consider here is also deterministic, but it differs from any of these previous models. Starting from some natural assumptions, we build a simple continuous-time model of traffic signals on a two-dimensional lattice; microcanonical ensembles are not considered, and elements are not chaotic. Interactions between neighbouring signals are realised naturally by the traffic flows between them, assuming that each signal is controlled on the basis of locally observed information on traffic flows. Probabilistic behaviour resembling the Ising model arises from these deterministic interactions.

## Results

### Traffic signal model

**Figure 1:**
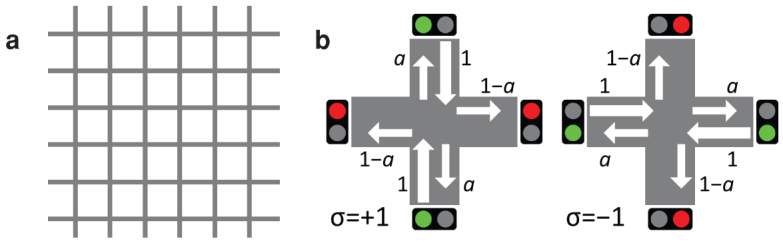
Schematic figures of the traffic signal model. (a) Grid pattern of roads. (b) The two states of traffic signals at each crossing.

Specifically, the model is formulated as follows. Traffic signals are located at every crossing (node) of a two-dimensional lattice (Fig. 1a) of size *L* × *L* with a periodic boundary condition. Roads exist between neighbouring nodes, and each road consists of two lanes, one in each direction. We regard vehicles as fluids that flow along the lanes; they flow into a traffic lane at a crossing and flow out at a neighbouring crossing. As shown in Fig. 1b, the signals at the *i*th node have one of the following two states: *σ_i_* = +1, in which vertical (north–south) traffic flow is allowed, and *σ_i_* = −1, in which horizontal (east–west) traffic flow is allowed. We assume that when *σ_i_* = +1, only the vehicles coming from the vertical lanes flow into the crossing at the rate of 1 (vehicle) per unit time; at the crossing, vehicles go straight at the rate of *a*, and they turn right or left at the rate of 1 − *a*, where *a* ∈ [0, 1] is a parameter of the model. Similarly, when *σ_i_* = −1, vehicles from the horizontal lanes are allowed to enter the crossing, where they behave in the same way.

Then, the number of vehicles *q_ij_* in the traffic lane from node *j* to node *i* evolves according to the following differential equation: 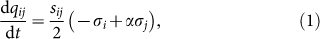
where *α* stands for 2*a* − 1, and *s_ij_* ∈ {+1, −1} denotes the direction of the lane from node *j* to node *i* (+1 for vertical and −1 for horizontal).

Traffic signals are controlled locally; essentially, they are changed to allow traffic from the lanes in which more vehicles are waiting at each crossing. This type of dynamic and decentralised control strategies, rather than conventional cyclic and centralised ones, has recently been considered as promising^1^,^2^,^3^,^4^. Let us consider the average numbers of vehicles coming from the vertical and horizontal lanes at the *i*th node. Their difference is given by 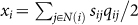, where *N*(*i*) denotes the set of four neighbours of the *i*th node. When *x_i_* > 0, more vehicles are waiting in the vertical direction, thus the vertical signals should be green (*σ_i_* = +1), and when *x_i_* < 0, the horizontal signals should be green (*σ_i_* = −1). To avoid frequent switching, signals are controlled by the following on–off control rule: 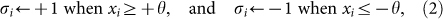
where *θ* > 0 is the threshold parameter for the deadband, and we set *θ* = 1 without loss of generality in the following.

Equations (1) and (2) define the dynamics of the traffic signal model. However, instead of considering the numbers of vehicles *q_ij_* in all the lanes, it is sufficient to consider only the differences *x_i_* at all the nodes. The time evolution of *x_i_* is described by 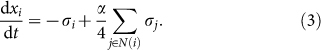
Note that because −1 ≤ *α* ≤ 1, *x_i_* never increases when *σ_i_* = +1, and never decreases when *σ_i_* = −1. Therefore, *x_i_* can be controlled within the interval [−*θ*, +*θ*]. This also means that the values of *q_ij_* are bounded and ensures that the lanes become neither too much congested nor empty provided that there are sufficient vehicles at the initial time. Because the traffic flows are controlled by discrete signals, the model can be regarded as a switched flow system^2^,^19^. More generally, it is a hybrid dynamical system^20^, because it is a completely deterministic system that consists of both continuous variables {*x_i_*} and discrete variables {*σ_i_*}.

The state (*x*_1_, …, *x_N_*) of *N* = *L* × *L* crossings moves straight in the *N*-dimensional hypercube [−*θ*, +*θ*]*^N^* according to equation (3), and its direction changes only on the boundary of the hypercube according to equation (2). Therefore, its dynamics can be understood as a pseudo billiard^21^ or a strange billiard^22^,^23^. Note that the dynamics is invertible. For *a* = 1/2 (i.e., *α* = 0), interactions between the *x_i_*'s vanish, and the dynamics reduces to a normal hypercubic billiard. The decoupled dynamics of the nodes can be considered as non-chaotic oscillators because they behave periodically with a period of 4*θ*.

### Ising-like dynamics

**Figure 2:**
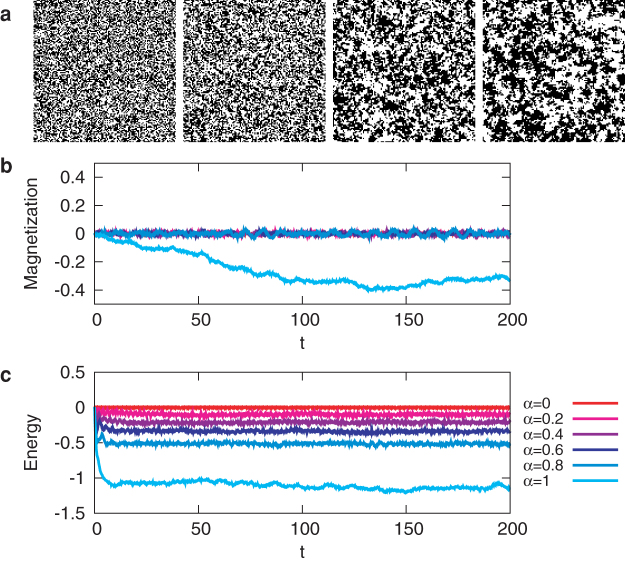
Typical dynamics of the traffic signal model. (a) Snapshots of traffic signals for *α* = 0, 0.5, 0.95, and 0.995 (from left to right). Black and white dots represent vertical and horizontal directions allowed at each crossing, respectively. (b, c) Time evolutions of (b) the magnetisation and (c) the energy per site for some values of *α* in a lattice of size *L* = 128. Initial values of *x_i_* and spin configurations *σ_i_* at time 0 are chosen from the uniform distribution on [−1, +1]*^N^* × {−1, +1}*^N^* (the high-temperature initial condition).

Typical dynamics of the model is shown in Fig. 2. The snapshots of the spin configurations of the traffic signal model (Fig. 2a) resemble those of the Ising model in the paramagnetic phase. Figures 2b and c show the time evolutions of the magnetisation per site, 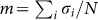, and the energy per site, 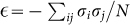, respectively, for several values of *α*. Apparently the system shows probabilistic behaviour, although each element is non-chaotic.

Neighbouring spins have ferromagnetic interactions for *α* > 0, since as equation (3) shows, *x_i_* moves more slowly when more neighbouring spins are aligned. Similarly, they have antiferromagnetic interactions for *α* < 0, and the model is symmetric with respect to *α* = 0 (see Methods for mathematical properties of the model). The parameter *α* corresponds to the coupling strength between the signals, which controls the apparent temperature of the system; as *α* goes away from 0, the system is considered to be cooled down. As *α* approaches 1, the average cluster size grows (Fig. 2a) and the average energy decreases (Fig. 2c).

**Figure 3:**
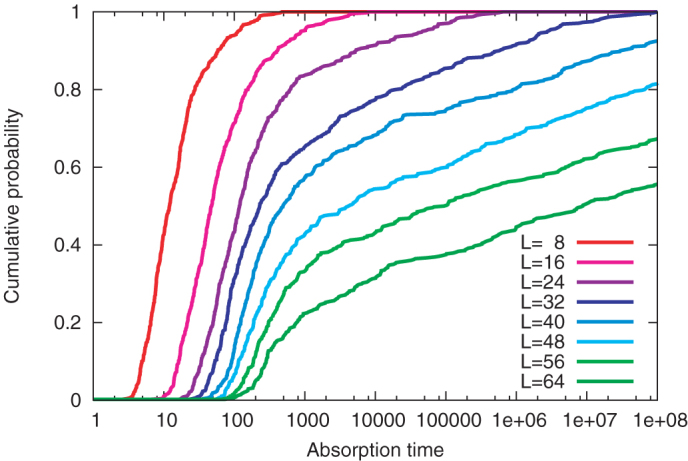
Relaxation time of the model for *α* = 1. Cumulative probabilities (the vertical axis) of absorption time (the horizontal axis, in logarithmic scale) are shown. For each *L*, the absorption times were calculated for 480 different high-temperature initial values. As *L* increases, the absorption time increases exponentially.

In the extreme case of *α* = 1, the system dynamics has two absorbing states; as equation (3) shows, once all the spins are aligned, the state never changes, and the system freezes. Figure 3 shows the time required to reach one of the absorbing states starting from a random (high-temperature) initial state. In all the calculations for *L* ≤ 32, the system dynamics reaches the absorbing states in a finite time *t* ≤ 10^8^. This implies that the traffic signal model of finite size undergoes spontaneous symmetry breaking as it approaches *α* = 1. However, the absorption time is likely to diverge in the thermodynamic limit, because in Fig. 3 the absorption time seems to increase exponentially as *L* increases.

**Figure 4:**
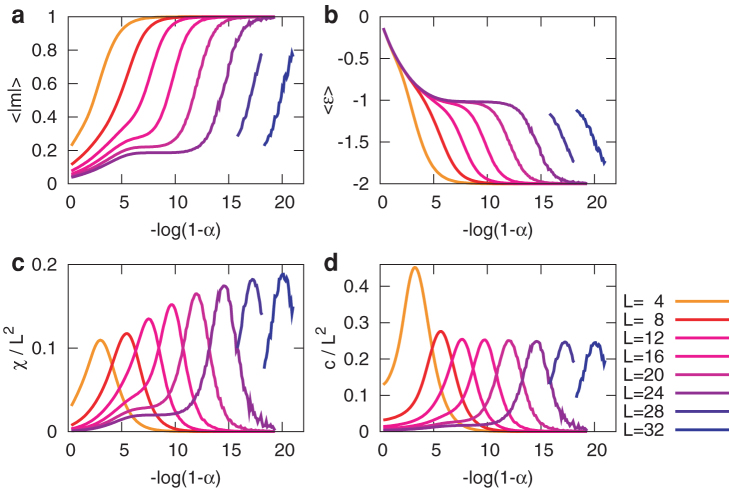
Critical behaviour of the model. The horizontal axis denotes −log(1 − *α*). For *L* ≤ 24, the statistics were calculated for 240 different high-temperature initial values during 10^6^ unit time after the initial 10^6^ unit time is skipped. For *L* = 28 and 32, only the statistics around the peaks are calculated for 120 initial values during 10^8^ unit time after 10^8^ unit time is skipped. (a) Average absolute magnetisation per site 〈|*m*|〉. (b) Average energy per site 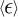. (c) Magnetic susceptibility per site *χ* divided by *L*^2^. (d) Specific heat per site *c* divided by *L*^2^.

Thermodynamic behaviour of the model as it approaches *α* = 1 is shown in Fig. 4. For |*α*| < 1, no absorbing states exist, and similarly to the case of the Ising model, it can be shown that the long-term average of the magnetisation 〈*m*〉 is strictly zero (see Methods). As *α* approaches 1, the average absolute magnetisation 〈|*m*|〉 increases from 0 to 1 (Fig. 4a), which suggests a phase transition from the paramagnetic phase to the ferromagnetic phase. In the corresponding range of *α*, the magnetic susceptibility *χ* = *N*(〈*m*^2^〉 − 〈|*m*|〉^2^) and the specific heat 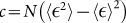 have peaks (Fig. 4c and d). The phase transition point indicated by these peaks seems to converge to *α* = 1 in the thermodynamic limit. This implies that the ordered behaviour exists only at *α* = 1 in the thermodynamic limit. In this sense, this phase transition in the model can be observed only as a finite size effect.

More detailed analysis of larger systems is necessary for accurate estimation of the critical exponents. However, because the calculation of thermodynamic quantities of the model requires lengthy computation, these analyses do not seem tractable at present.

The thermodynamic behaviour is realised by nonlinear dynamics of coupled non-chaotic elements. The dynamics is not ergodic in a rigorous sense, because it can be shown that it has invariance (see Methods). However, this non-ergodicity may be subtle from a macroscopic point of view. The dynamics can be characterised by the piecewise linear Poincaré map defined on the boundary of the hypercube, which we call the spin-flip map (see Methods).

**Figure 5:**
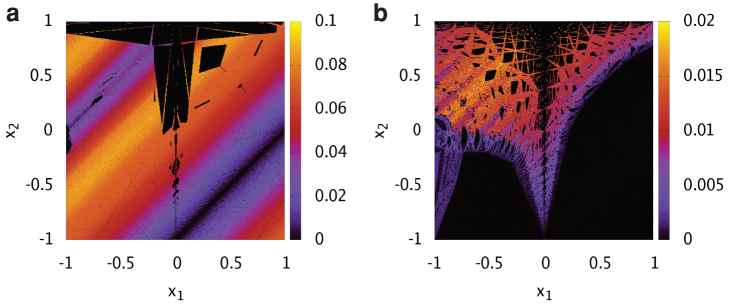
Traffic signal model of size *L* = 2. Complicated structures in the state space for (a) *α* = 0.8 and (b) *α* = 0.2 are shown. Colours represent the largest Lyapunov exponents of the spin-flip map calculated for 10^5^ iterations starting from the initial values ***x***(0) = (*x*_1_, *x*_2_, 0, 1) and ***σ***(0) = (+1, +1, +1, +1). Lines in the diagonal direction (*x*_1_ − *x*_2_ = const.) correspond to invariant planes. The largest Lyapunov exponents vary depending on the invariant plane. Even on the same invariant plane, non-chaotic (black) and chaotic (coloured) regions coexist. The dynamics depends strongly on the choice of initial values.

We numerically investigated the dynamics for the smallest case *L* = 2. Figure 5 shows the largest Lyapunov exponents of the spin-flip map for initial values on a plane in the boundary. The largest Lyapunov exponents are non-negative. It can be observed that the instability of the orbits depends strongly on the invariant plane; namely, it depends on the choice of initial states. Moreover, even if we limit the dynamics to an invariant plane, there are typically multiple invariant sets (ergodic components). Therefore, even in the chaotic case, the state wanders in a limited region on the invariant plane. It should be investigated as a next step whether these properties for *L* = 2 remain in larger systems.

## Discussion

Chaotic dynamics is known to emerge even in a very simple switched flow system^19^. Therefore, it has been pointed out that city traffic as a switched flow system may exhibit chaotic behaviour^2^. In this regard, we have presented the first concrete traffic model described by a switched flow system that exhibits chaotic dynamics in this paper.

The probabilistic distribution of spin configurations realised by the chaotic behaviour is unknown and should be investigated in the future. However, it can be intuitively related to the Ising model. Let us consider the behaviour of the *i*th node in the lattice surrounded by four neighbouring nodes whose states are all fixed. Then according to Eq. (3), *x_i_* moves at the absolute speed of (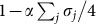) when *σ_i_* = +1, and (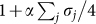) when *σ_i_* = −1. Therefore, if we sample *σ_i_* at a random time, the probability of observing *σ_i_* = +1 is given by 
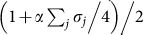
, which is determined by the states of the neighbouring nodes as in the Ising model. This intuitive discussion gives us a probabilistic Ising-like model that may be approximately followed by the determinstic model.

Because the traffic signal model is very simple, it can be extended in many directions and related to studies in various fields. External magnetic fields can easily be implemented by considering different speeds of flows in the horizontal and vertical directions. Extensions to a *d*-dimensional hyperlattice and other network structures can be considered by modifying equation (3). The traffic signal model can also be regarded as a coupled oscillator system in which, unlike the Kuramoto model^24^, the elements interact only through binarised phases. In the sense that interactions are discrete, the model may be related to neural networks; in fact, it is similar to a neural network model of simplified hysteresis neurons^25^.

Equation (3) can be generalised to the form 
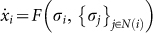
, which in conjunction with equation (2) defines a new class of dynamical systems with a large number of degrees of freedom. This new class is expected to exhibit chaotic pseudo-billiard dynamics that arises from coupled non-chaotic elements. Beyond what is shown in this paper, it may have connections to various models such as spin models in statistical physics, coupled oscillators and CMLs in nonlinear physics, and cellular automata in computer science. Recently, we have shown that the Boltzmann machines can be simulated by chaotic dynamics in this class^26^.

It should be also noted that over the past half-century, ideas from statistical physics have been successfully applied to traffic flows^27^. In particular, cellular automaton models of city traffic such as the BML model^28^ and the CS model^29^ are built on a similar configuration of two-dimensional lattices. However, while these studies focused on vehicles and characterised traffic jams as phase transitions, we focused on traffic signals, which themselves constitute a many-body system in cities. Although the model presented here is too simple for real city traffic, it is natural to assume that signals are interacting with each other if they are somehow controlled locally, as pointed out in the previous studies^1^,^2^. In this sense, our model is expected to serve as a starting point for considering statistical mechanics of traffic signals.

## Methods

### Symmetries in the model

To understand the dynamics of the model, it is important to understand symmetries inherent in the model. The time evolution of *x_i_*'s (Eq. (3)) can be written in a matrix form as follows: 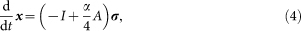
where 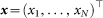, 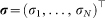, and the matrix *A* is the adjacency matrix of the lattice. There is a trivial symmetry in the state space of the nodes as follows: 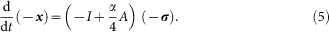
There is also a symmetry as to time reversal as follows: 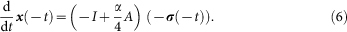
Let *L*_+_ and *L*_−_ be the sets of the black and white sites in the checkerboard pattern on the lattice. Define *D* = diag(*d*_1_, …, *d_N_*), where *d_i_* = +1 if *i* ∈ *L*_+_ and *d_i_* = −1 if *i* ∈ *L*_−_. Since *DA* = −*AD*, we have 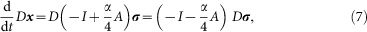
which shows the symmetry between ferromagnetism and antiferromagnetism (+*α* and −*α*).

### Long-term average of the magnetisation

In the traffic signal model, it can be shown that the long-term average of the magnetisation is strictly zero, as in the Ising model on a finite-size lattice. By averaging the system dynamics from time 0 to *T*, we have 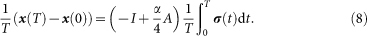
In the limit *T* → ∞, the left-hand side of this equation goes to zero, because 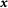 is bounded. For |*α*| < 1, the matrix (−*I* + (*α*/4)*A*) is diagonal dominant and regular, so that 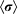 is zero. Therefore, the long-term average of the magnetisation 〈*m*〉 must be also zero.

### Invariant hyperplanes

As one of the dynamical properties of the model, it can be shown that the system is not ergodic. Let us define Γ as follows: 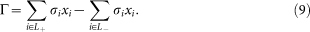
While there is no spin flips, the value of Γ does not change, because 
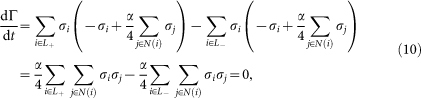
where *N*(*i*) denotes the set of four neighbouring nodes of node *i*. When a spin flips, the value of Γ changes only by multiples of two. When *σ_i_* changes from +1 to −1, *x_i_* has to be +1, and similarly when *σ_i_* changes from −1 to +1, *x_i_* has to be −1. Therefore, *σ_i_x_i_* always changes from +1 to −1 when the *i*th spin flips. Therefore the system evolves within the invariant hyperplanes determined by the invariance of Γ modulo 2.

### Spin-flip map

To analyse the dynamics of the model, it is important to analyse the Poincaré map defined on the boundary of the hypercube, which we call the spin-flip map. Let 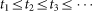 be the time sequence of spin flips. For convenience, define 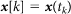 and 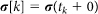. The *k*th time interval *τ_k_* = *t_k_*_+1_ − *t_k_* of the spin sequence is given by 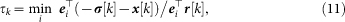
where ***e****_i_* is the *i*th standard basis for *N*-dimensional Euclidean space and 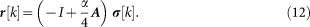
Let *i*[*k*] be the spin actually flipped at time *t_k_*. Then, we have the spin-flip map 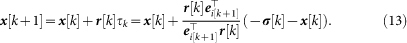
Therefore, the spin-flip map is piecewise linear.

## Author Contributions

H.S. and J.I. developed the model, and H.S. analysed it. H.S., J.I. and K.A. designed the research, and wrote and reviewed the manuscript.
